# Evaluation of an open access echocardiography service in the Netherlands: a mixed methods study of indications, outcomes, patient management and trends

**DOI:** 10.1186/1472-6963-10-37

**Published:** 2010-02-10

**Authors:** Leanne MSG van Heur, Leo HB Baur, Marleen Tent, Cara LB Lodewijks-van der Bolt, Marjolijn Streppel, Ron AG Winkens, Henri EJH Stoffers

**Affiliations:** 1 Maastricht University Medical Centre, CAPHRI School for Public Health and Primary Care, Department of General Practice, PO Box 616, 6200MD, Maastricht, The Netherlands; 2 Atrium Medisch Centrum Parkstad, Department of Cardiology, PO Box 4446, 6401CX, Heerlen, The Netherlands; 3 Maastricht University Medical Centre, Department of Integrated Care, PO Box 5800, 6202AZ, Maastricht, The Netherlands; 4 Streekziekenhuis Koningin Beatrix, PO Box 9005, 7100GG Winterswijk, The Netherlands

## Abstract

**Background:**

In our region (Eastern South Limburg, The Netherlands) an open access echocardiography service started in 2002. It was the first service of this kind in The Netherlands. Our study aims were: (1) to evaluate demand for the service, participation, indications, echocardiography outcomes, and management by the general practitioner (GP); (2) to analyse changes in indications and outcomes over the years.

**Methods:**

(1) Data from GP request forms, echocardiography reports and a retrospective GP questionnaire on management (response rate 83%) of 625 consecutive patients (Dec. 2002 - March 2007) were analysed cross-sectionally. (2) For the analysis of changes over the years, data from GP request forms and echocardiography reports of the first and last 250 patients that visited the service between Dec. 2002 and Feb. 2008 (n = 1001) were compared.

**Results:**

The echocardiography service was used by 81% of the regional GPs. On average, a GP referred one patient per year to the service. Intended indications for the service were dyspnoea (32%), cardiac murmur (59%), and peripheral oedema (17%). Of the other indications (22%), one-third was for evaluation of suspected left ventricular hypertrophy (LVH). Expected outcomes were left ventricular dysfunction (LVD) (43%, predominantly diastolic) and valve disease (25%). We also found a high proportion of LVH (50%). Only 24% of all echocardiograms showed no relevant disease. The GP followed the cardiologist's advice to refer the patient for further evaluation in 71%. In recent patients, more echocardiography requests were done for 'cardiac murmur' and 'other' indications, but less for 'dyspnoea'. The proportions of patients with LVD, LVH and valve disease decreased and the proportion of patients with no relevant disease increased. The number of advices by the cardiologists increased.

**Conclusion:**

Overall, GPs used the open access echocardiography service efficiently (i.e. with a high chance of finding relevant pathology), but efficiency decreased slightly over the years. To meet the needs of the GPs, indications might be widened with 'suspicion LVH'. Further specification of the indications for open access echocardiography - by defining a stepwise diagnostic approach including ECG and (NT-pro)BNP - might improve the service.

## Background

Heart failure is a progressive disease with a high morbidity and mortality that affects roughly 2-3% of the Western Population [1]. In individuals aged 55, almost one-third will develop heart failure during their remaining lifespan. Although prognosis has improved due to better treatment options, only 50% of all patients are still alive four years after the initial diagnosis [1]. Several studies have shown that patients thought to have heart failure frequently have been misdiagnosed [2,3]. Without an accurate diagnosis, many patients will be treated inappropriately [4]. Heart failure is difficult to diagnose, especially in the early stages of the disease. Symptoms and signs are important in suggesting heart failure, but they are not sufficiently specific for establishing the diagnosis [5]. Therefore, a patient with suspected heart failure must have objective tests to confirm the diagnosis. To date, the gold standard to establish the diagnosis is an echocardiogram [1]. In countries where the general practitioner (GP) has the role of being gate-keeper for specialist care, this would require a referral to a cardiologist.

In the United Kingdom several studies were conducted to evaluate open access echocardiography services [6,7]. In these studies, GPs appraised the open access service positively. Furthermore, the echocardiography requests from primary care did not overload the echocardiography department of the hospital. Inspired by these experiences, cardiologists in our region (around the city of Heerlen, in the south of The Netherlands) started an open access echocardiography service in 2002. It was the first service of this kind in The Netherlands. The idea was to lower the threshold for GPs for supplementary diagnostic testing in patients with a raised suspicion of heart failure. Until then, a referral to the cardiologist was required. Our group performed a pilot study in two primary health care centres in the Heerlen region, to explore the feasibility of open access echocardiography. Subsequently, the service was extended to all GPs in our region [8].

The aim of the present study was to evaluate the service with regard to participation level of GPs in our region, indications for an echocardiography request, outcomes of the echocardiograms, advice given to the GPs by the cardiologists and management of the GP after having received the advice. Additionally, we wanted to analyse changes in indications and outcomes over the years. Thus, we hoped to obtain suggestions for improvement of the service.

## Methods

### The open access echocardiography service

The service started as a pilot project for two primary health care centres with 13 GPs in December 2002. After a positive evaluation, all GPs in Eastern South Limburg (ESL) were invited to use the service. This region, with approximately 240.000 inhabitants, is an urban area around the city of Heerlen, located in the south of The Netherlands. It has one hospital organisation with three locations. There were 129 GPs working in 71 practices.

GPs were given the opportunity to order an echocardiogram via the hospital's diagnostic centre without referring the patient to a cardiologist, when they suspected the patient of having heart failure or valve disease. The GPs received a short training on the indications and restrictions for echocardiography and on the interpretation of the results. To prevent an overload of requests, the diagnostic indications were restricted to dyspnoea, cardiac murmur and peripheral oedema. It was agreed that the cardiologist would summarize the results and add an advice for the GP.

Following the request, the echocardiogram was made at the department of diagnostic imaging at the regional hospital (Atrium Medisch Centrum Parkstad, Heerlen). The results from the echocardiogram were interpreted by the cardiologist and sent to the GP. If the echocardiogram showed relevant abnormalities, the GP was advised to refer the patient to the cardiologist, to start or change medication, to repeat the echocardiogram within a few years or to consider endocarditis prophylaxis. The cardiologist could give more than one advice (i.e. 'repeat the echocardiogram within a few years' and 'consider endocarditis prophylaxis'). Adherence to the advice was left to the GP.

### Study design

Data for evaluation of GP participation level, echocardiography indications, echocardiography outcomes, and cardiologist's advice were collected in consecutive patients and analysed cross-sectionally. Data of each patient referred to the open access echocardiography service were automatically included in the study, unless patients did not give their consent. Consent was asked when patients visited the echocardiography service. Data on GP management after the echocardiogram, were collected retrospectively in the electronic medical record system of the referring GPs, and analysed cross-sectionally. Changes in indications, outcomes and advice over the years were assessed by a quasi-longitudinal comparison of two independent samples of consecutive patients.

The medical ethics committee of the Atrium Medical Centre (METC Atrium-Orbis-Zuyd) had approved the study (METC number 08-N-15; Nederlands Trial Register NTR1231).

### Data collection for this study

#### (1) Consecutive data collection

##### Echocardiography request form

A short request form was created for the GPs with tick boxes for quick completion. The GPs had to fill in the indication for ordering an echocardiogram, current medication (diuretic, nitrate, RAAS-inhibitor, etc.), relevant medical history (hypertension, diabetes, coronary insufficiency, obesity, COPD) and the findings of physical examination (blood pressure, heart rate, cardiac murmur, raised jugular vein pressure and peripheral oedema). The data from the request forms were registered anonymously in an SPSS database (MT, MS, LvH).

##### Echocardiography reports

The echocardiograms were performed by (one of) four cardiologists with experience in cardiac imaging or a resident supervised by one of these cardiologists (Philips SONOS 5500 system, ENCONCERT digital storage and retrieval system). The results were interpreted by the cardiologist according to the criteria of the American and European Societies of Echocardiography [9-12]. The following definitions were used:

###### - Left ventricular dysfunction (LVD)

Systolic LVD was defined as a left ventricular ejection fraction <40%. Diastolic dysfunction was estimated using peak velocity E/A ratios adjusted for age. Per age category cut-off values of peak velocity E/A ratio were used to estimate diastolic dysfunction [13]. Because for patients younger than 20 years of age as yet no reliable cut-off values have been described in literature, the assumption was made that these patients had no diastolic dysfunction.

###### - Valve disease

Aortic insufficiency was measured using the criteria of Perry and Reynolds and was assumed to be important if it was grade 2 or more [14,15]. Aortic valve stenosis was considered important if the maximal gradient was ≥ 30 mmHg or the mean gradient ≥ 20 mmHg. Aortic valve disease was defined as the presence of important aortic valve insufficiency, important aortic valve stenosis or a bicuspid aortic valve. Mitral valve insufficiency was graded by measurement of the jet area and proximal jet width at the vena contracta in addition to measurement of the continuous wave flow and the pulsed wave flow in the pulmonary veins. Mitral valve insufficiency was assumed to be important if leakage was grade 2 or more. Mitral valve stenosis was considered important when the maximal gradient was ≥ 10 mmHg. Mitral valve disease was defined as the presence of important mitral valve insufficiency, important mitral valve stenosis or a mitral valve prolaps. Pulmonary valve insufficiency was assumed to be important if the leakage was grade 2 or more. Pulmonary valve stenosis was considered important if the maximal gradient was ≥ 15 mmHg. Pulmonary valve disease was defined as the presence of important pulmonary stenosis or insufficiency. Tricuspid insufficiency was assumed to be important if the leakage was grade 2 or more.

###### - Other abnormalities

Left ventricular hypertrophy (LVH) was defined as a left ventricular mass calculated with the method of Devereux and indexed for length exceeding 102 g/m for males and 143 g/m for females [16]. Pulmonary hypertension was considered present if the measured systolic pulmonary pressure was ≥ 35 mmHg. Measurement of the systolic pulmonary pressure was done by measuring the maximal velocity of the tricuspid regurgitation and calculation of the systolic pressure gradient between the right ventricle and right atrium according to the Bernoulli equation. Right atrial pressure was estimated by looking to the diameter and the collapse of the inferior cava vein. The septum was examined for defects such as persistent foramen ovale and atrial septum defect.

###### - No disease

In case there were no abnormalities, i.e. neither LVD, nor valve disease, LVH, pulmonary hypertension nor a septum defect, the outcome was coded as 'no disease'.

All data from the echocardiography reports were collected and stored anonymously in an SPSS database (MT, MS, LvH). In case of the absence of comments about previously mentioned structures and functions in echocardiographic reports, the assumption was made that there were no relevant abnormalities.

#### (2) Retrospective survey on management by the GP

The results of the ultrasound Doppler examination were returned to the GP with a comment on how to manage the patient. Between December 2002 and March 2007, 625 consecutive patients had visited the open access echocardiography service. In April 2007, all GPs who had participated were sent one or more questionnaires, depending on the number of patients for whom they had ordered an echocardiogram. It was a short questionnaire, almost fitting on one A4-page, containing five multiple choice questions with multiple answer options, on: (1) the GP's considerations to order an echocardiogram, (2) the diagnostic procedures performed before the ordering of the echocardiogram, (3) known morbidity at that time, (4) medication use at that time, and (5) actions of the GP after the echocardiography result had been received (i.e. changes in cardiovascular or pulmonary medication, and other actions including referral to cardiologist or other medical specialist) (see additional file 1). In an accompanying letter, for each patient the date on which the echocardiogram was made was given. The GP was instructed to check the medical record of each patient, i.e. to locate the echocardiogram result and find the requested information on echogram ordering (before echocardiography) and patient management (after echocardiography), respectively.

We aimed for a minimal response rate of 60%. After a first mailing (April 2007), a second mailing (October 2007) was sent to all non-responders. After that, a reminder was done by e-mail and telephone to the non-responding GPs. The data from the questionnaire were collected and stored anonymously in an SPSS database (MT, LvH). Data were missing due to non-returned questionnaires or death of the patient (6).

### Statistical analysis

Statistical analysis was done with SPSS for Windows version 13.0. Data from request forms, echocardiography reports and questionnaires were coded and variables were described. New values for variables were computed, based on numeric transformations of the existing variables.

Data for evaluation of GP participation level, echocardiography indications, echocardiography outcomes, and cardiologists' advice were analysed cross-sectionally, as were the data on GP management after the echocardiogram. For description of the different variables, frequency tables were made.

After these cross-sectional analyses had been done, we thought it would be worthwhile to study trends in indications and outcome. At that time (February 2008), the database had increased to 1001 patients, providing the opportunity for a quasi-longitudinal comparison of two independent samples of consecutive patients. We decided to compare the first and last 250 patients who had visited the echocardiography service between December 2002 and February 2008, for indications, echocardiographic outcomes and advice given. Therefore, the database was sorted according to date of investigation and the first and last 250 patients were selected, respectively. For comparison of percentages, the chi-square test was used. Continuous variables were analyzed using the independent samples T-test.

## Results

### Use of the open access service by GPs

During a study period of 4.5 years, a request for echocardiography was made for 625 patients by 118 GPs from 70 practices, including 13 GPs from outside the region. This implies an average of five patients per participating GP, one patient per GP per year (range 0-9). The participation rate of GPs from the ESL region was 81.4% (105/129).

### Patient characteristics

Of the patients included, 365 (58%) were female and 260 (42%) were male. Mean age was 60.5 years (SD: 18.2 years); 59.2 years (SD: 16.7 years) for men and 61.4 years (SD: 19.1 years) for women. Of the patients of whom the GP returned the questionnaire, 61.9% (320/517) had at least one relevant disease in their medical history. The most frequently reported disorder was hypertension (241; 46.6%).

### Indications for echocardiography requests (Table 1)

**Table 1:** General practitioner's indications for open access echocardiography

Indications for an echocardiography request	Frequency	Percentage*
*Single indications*		
Cardiac murmur only	243	38.9
Dyspnoea only	78	12.5
Peripheral oedema only	24	3.8
'Other' only	102	16.3
*Combined indications*		
Dyspnoea and cardiac murmur	59	9.4
Dyspnoea and peripheral oedema	41	6.6
Cardiac murmur and other	29	4.6
Cardiac murmur and peripheral oedema	20	3.2
Dyspnoea, cardiac murmur and peripheral oedema	17	2.7
Dyspnoea and other	3	0.5
Peripheral oedema and other	3	0.5
		
*No indication given*	6	1.0

* Total population: n = 625

The reasons for an echocardiography request by the GP were distributed as follows: cardiac murmur 368 (58.9%), dyspnoea 198 (31.7%), and peripheral oedema 105 (16.8%). No indication was given in six patients (1%). Details are presented in Table 1. The group of patients with other indications than was agreed upon beforehand, was rather large (137, 21.9%). The most common 'other' indication for an echocardiography request was evaluation of LVH, based on ECG abnormalities or the presence of hypertension (42, being 30.7% of the 'other' indications).

### Outcome of the echocardiograms (Table 2)

**Table 2:** Outcomes of echocardiography ordered by general practitioners (%)

GPs' indications for an echocardiography request		Echocardiographic outcome							
		**Left ventricular dysfunction**				**Valve disease**	**LVH**	**PHT**	No disease*
		***Systolic***	***Diastolic***	***Both***	***Any***				

**Cardiac murmur **(total) n = 368		13 (3.5)	142 (38.6)	7 (1.9)	148 (40.2)	115 (31.3)	190 (51.6)	56 (15.2)	87 (23.6)
**Dyspnoea **(total) n = 198		21 (10.6)	73 36.9)	9 (4.5)	85 (42.9)	46 (23.2)	107 (54.0)	37 (18.7)	42 (21.2)
**Peripheral oedema **(total) n = 105		15 (14.3)	42 (40.0)	7 (6.7)	50 (47.6)	29 (27.6)	55 (52.4)	25 (23.8)	17 (16.2)
**Other **(total) n = 131 (100%)		3 (2.2)	68 (49.6)	3 (2.2)	68 (49.6)	20 (14.6)	60 (43.8)	15 (10.9)	31 (22.6)
*Hypertension, LVH?* n = 42 (30.7%)		1 (2.4)	25 (59.5)	1 (2.4)	25 (59.5)	6 (14.3)	23 (54.8)	6 (14.3)	8 (19.0)
*Non-specific Complaints* n = 20 (14.6%)		1 (5.0)	9 (45.0)	1 (5.0)	9 (45.0)	7 (35.0)	7 (35.0)	2 (10.0)	6 (30.0)
*ECG abnormalities* n = 19 (13.9%)		0 (0)	11 (57.9)	0 (0)	11 (57.9)	0 (0)	2 (10.5)	2 (10.5)	7 (36.8)
*Arrhythmias* n = 17 (12.4%)		0 (0)	8 (47.1)	0 (0)	8 (47.1)	2 (11.8)	8 (47.1)	0 (0)	2 (11.8)
*Septum defects* n = 11 (8.0%)		0 (0)	5 (45.5)	0 (0)	5 (45.5)	0 (0)	4 (36.4)	0 (0)	4 (36.4)
*Cardiac Diseases *in Family n = 8 (5.8%)		0 (0)	2 (25.0)	0 (0)	2 (25.0)	1 (12.5)	3 (37.5)	0 (0)	3 (37.5)
*Other* n = 20 (14.6%)		1 (5.0)	8 (40.0)	1 (5.0)	8 (40.0)	4 (20.0)	13 (65.0)	5 (25.0)	1 (5.0)

Abbreviations: GP general practitioner; LVH left ventricular hypertrophy; PHT pulmonary hypertension

* No disease: neither valve disease nor left ventricular dysfunction or left ventricular hypertrophy or pulmonary hypertension or septum defects

The most frequent echocardiographic diagnoses were LVH (313, 50.1%), diastolic LVD (236, 37.8%) and 'any valve disease' (155, 24.8%). Pulmonary hypertension was present in 93 patients (14.9%). LVH and diastolic LVD were present in high percentages in each indication group. A negative echocardiography outcome (i.e. no relevant disease) was present in 147 patients (23.5%). Further details are presented in Table 2.

Table 2 also describes the detailed outcomes for the indication 'other'. On average, the percentage of patients without abnormalities in this group (22.6%) was similar to that of the original indications. The percentage of LVH in patients tested for that particular reason was high (23/42, 54.8%). In addition, the presence of LVD was high in this subgroup (25/42, 59.5%).

### Advice of cardiologist to GP (Table 3)

**Table 3:** Advice given by the cardiologist, in percentages

Advice given	Patients *with *any disease* n = 478	Patients *without *any disease n = 147	Total population n = 625
**No advice**	52.5	83.7	59.8
Advice**	47.5	16.3	40.2
*Refer to cardiologist*	*28.7*	*7.5*	*23.7*
*Repeat echocardiogram in x years*	*11.5*	*5.4*	*10.1*
*Endocarditis prophylaxis*	*13.8*	*4.8*	*11.7*
*Adjust medication*	*3.1*	*2.7*	*3.0*
*Refer to pulmonologist*	*0.8*	-	*0.6*
*Wrong indication for referral*	*4.8*	*1.4*	*4.0*

*Any disease: at least one relevant abnormality, i.e. valve disease or left ventricular dysfunction or left ventricular hypertrophy or pulmonary hypertension or septum defects

** The cardiologist could give more than one advice (i.e. 'Repeat echocardiogram in x years' and 'Endocarditis prophylaxis'); therefore, the sub-percentages of 'Advice' should not be summed.

Of all echocardiography reports, 251 (40.2%) contained a specific advice to the GP. Details are given in Table 3. For the group of patients with at least one echocardiographic abnormality (n = 478), this percentage was 47.5%. In patients with an echocardiographic abnormality, the advice to refer the patient to a cardiologist was given in 28.7%.

### Management by the GP (Table 4)

**Table 4:** Management by general practitioners after open access echocardiography (%)

Indication	A:	B:	Management by GP	
	**Patients with a relevant echocardiographic** diagnosis*, of whom the GP returned the questionnaire on management	**Advice of cardiologist to refer patient** ***(% of A)***	***GP *followed *cardiologist's advice to refer patient*** ***(% of B)***	***GP referred patient *without advice *of cardiologist*** ***(% of [A-B])***

**Whole group** n = 625	397	114 (28.7)	81 (71.1)	47 (16.6)
**Cardiac murmur** n = 368	237	73 (30.8)	56 (76.7)	26 (15.9)
**Dyspnoea** n = 198	128	43 (33.6)	30 (69.8)	16 (18.8)
**Peripheral oedema** n = 105	75	35 (46.7)	27 (77.1)	12 (30.0)
**Other** n = 137	84	19 (22.6)	8 (42.1)	12 (18.5)

Abbreviations: GP general practitioner; LVD left ventricular dysfunction

*Relevant Echocardiographic Diagnosis: at least one relevant abnormality, i.e. valve disease or left ventricular dysfunction or left ventricular hypertrophy or pulmonary hypertension or septum defects

The response rate of the GP questionnaire on patient management was 82.7% (517/625). Of 397 patients with at least one echocardiographic abnormality, a questionnaire on management was available. For 114 (28.7%) of these patients, the GP received the advice to refer the patient to the cardiology outpatient department. Such an advice was received relatively often when the indication for the echocardiography had been peripheral oedema (see Table 4), or when the outcome of the echocardiographic examination was valve disease (54.4%).

In 81 cases (71.1%), the GP followed the advice of the cardiologist and indeed referred the patient to the outpatient department (Table 4). This was done relatively often, if the indication for the echocardiography had been peripheral oedema or cardiac murmur. In the indication group 'other', GPs received relatively few advices for referral to the cardiologist compared with the original indication groups. In addition, the follow-up of a referral advice was relatively low in this group.

Of all patients with at least one echocardiographic abnormality but without an advice for referral, the GP still referred 47 patients (16.6%) to the cardiology outpatient department. This was done relatively often in case of an 'other' indication with established valve disease (37.5%), 'peripheral oedema' with established LVD (28.6%) or valve disease (30.4%), a cardiac murmur with established valve disease (28.2%), and dyspnoea with established LVD (25.6%).

Of all 517 patients of whom GP management data were available, eventually 128 (24.8%) were referred to the cardiology outpatient department after the echocardiographic examination.

### Comparison of indications and outcome over the years (Table 5)

**Table 5:** Open access echocardiography: significant changes in indications, outcomes and specialist advice (SD, %)

Variable		First 250 patients	Last 250 patients	Change	p-value
***Patient characteristics***					
Mean age, in years (SD)		62.6 (18.0)	59.5 (19.1)		0.057
Gender, number of females		147 (58.8)	151 (60.4)		0.715

***Indications***					
Cardiac murmur only		79 (31.6)	100 (40.0)	**+**	0.050
Any dyspnoea		111 (44.4)	65 (26.0)	**-**	0.000
*Dyspnoea only*		50 (20.0)	20 (8.0)	**-**	0.000
*Dyspnoea and peripheral oedema*		24 (9.6)	11 (4.4)	**-**	0.023
'Other' indications		41 (16.4)	67 (26.8)	**+**	0.005
*'Other' only*		27 (10.8)	55 (22.0)	**+**	0.001
***Echocardiographic outcome***					
Any LVD		110 (44.0)	85 (34.0)	**-**	0.022
*Diastolic LVD only*		95 (38.0)	74 (29.6)	**-**	0.047
Any valve disease		65 (26.0)	43 (17.2)	**-**	0.017
*Mitral insufficiency*		18 (7.2)	8 (3.2)	**-**	0.044
*Pulmonary insufficiency*		7 (2.8)	0 (0)	**-**	0.008
*Tricuspid insufficiency*		21 (8.4)	3 (1.2)	**-**	0.000
LVH		121 (48.4)	99 (39.6)	**-**	0.047
PHT		48 (19.2)	23 (9.2)	**-**	0.001
No disease*		55 (22.0)	82 (32.8)	**+**	0.007
***Outcome per indication***					
PHT/cardiac murmur		24/131 (18.3)	11/147 (7.5)	**-**	0.007
PHT/dyspnoea		23/111 (20.7)	6/65 (9.2)	**-**	0.047
No disease/dyspnoea		23/111 (20.7)	23/65 (35.4)	**+**	0.033
No disease/'other'		6/41 (14.6)	22/67 (32.8)	**+**	0.036
***Cardiologist's advice in patients with positive echocardiographic outcome***					
Any advice		78/195 (40.0)	100/168 (59.5)	**+**	0.000
*Repeat echocardiogram in x years*		8/195 (4.1)	28/168 (16.7)	**+**	0.000
*Adjust medication*		8/195 (4.1)	21/168 (12.5)	**+**	0.003

Abbreviations: LVD left ventricular dysfunction; LVH left ventricular hypertrophy, PHT pulmonary hypertension; + increase; - decrease

*No disease: neither valve disease nor left ventricular dysfunction or left ventricular hypertrophy or pulmonary hypertension or septum defects

In Table 5 a comparison is given between the first and last 250 patients that used the open access echocardiography service between December 2002 and February 2008 (n = 1001). Both samples did not differ statistically significant with regard to mean age and gender. Table 5 shows only the statistically significant differences in indications and outcomes. In the most recent group more echocardiography requests were done for 'cardiac murmur' and 'other' indications, but less for 'dyspnoea', as compared to the first group. The proportion of patients with LVD decreased (34.0% vs. 44.0%) as did the proportion of patients with valve disease (17.2% vs. 26.0%) and LVH (39.6% vs. 48.4%). Conversely, the proportion of patients with no relevant echocardiographic abnormality increased (32.8% vs. 22%). As compared to earlier patients, in recent patients with echocardiographic abnormalities, the cardiologists gave more advice to the GPs (59.5% vs. 40.0%), especially on repeating the echocardiographic examination and adjustment of medication.

## Discussion

### Summary of main findings

The participation rate of GPs in the study region was 81%. Each participating GP referred roughly one patient per year to the open access echocardiography service. Only in less than a quarter of all cases, no echocardiographic abnormalities were found. An unintended outcome was the high proportion of patients with LVH (50%). One-fifth of all requests were for 'other' indications than was agreed upon beforehand. Evaluation of possible LVH was the most common indication in this group. More than half of these patients actually had LVH. In half of all patients with an echocardiographic abnormality, the GP received an advice of the cardiologist. Of all patients, eventually a quarter was referred to the cardiology outpatient department. Over a period of five years, the indications for an echocardiographic request shifted towards 'cardiac murmur' and 'other indications'. A decrease of relevant outcomes (LVH, LVD, valve disease) and an increase of 'no disease' were observed. Cardiologists improved in giving advice to the GPs.

### Strengths and limitations of the study

This study describes echocardiography data from primary care. Our analysis comprised a period of 4.5 to more than 5 years, using data collected during regular patient care. This allowed us to include a large number of cases that reflect daily general practice. However, in some echocardiography reports information was missing about certain structures or functions. In these cases, the assumption was made that there were no relevant abnormalities.

In this study, we used a retrospective questionnaire to acquire information about the management of the GPs. The questionnaire response rate was high (82.7%), indicating a low risk of selection bias. However, GPs had to fill in information about patients who sometimes were referred a few years ago; for questions about medication, additional diagnostic tests and medical history this will not have been a problem, because these data are clearly registered in the GP's electronic patient record. Nevertheless, we cannot rule out that in some cases the GP will have relied on his memory when he did not find the exact answer to a question in the patient record. We also could not correct for possible missing data in the medical records. This may have caused a negative bias in our results on the adherence of the GPs to the advice of the cardiologists.

Finally, we did neither estimate costs nor compare the cost-effectiveness of scenarios with and without the open access service, respectively. This would require an RCT design.

### Diastolic left ventricular dysfunction, left ventricular hypertrophy and hypertension

The intended outcomes of the echocardiograms were valve disease and LVD. We found a higher proportion of patients with LVD than has been reported in previous studies [17,18]. However, these studies only examined systolic dysfunction, whereas in our study diastolic dysfunction was also examined and in fact formed the majority of the LVD cases.

In our study the proportion of echocardiography requests for indications other than was agreed upon beforehand, was higher (22%) than we had expected from earlier studies [8,19]. One third of these requests were made by the GPs for evaluation of LVH. This suggests that GPs consider LVH evaluation a useful indication for open access echocardiography. The results showed that many of these patients (55%) indeed appeared to have LVH.

The high proportions of diastolic LVD and LVH reflect the importance of hypertension as cause of heart failure in primary care patients. This is in accordance with the study of Rutten et al, who compared cardiologists and GPs for what they considered important factors for the diagnosis of heart failure. For cardiologists the three most important cardiovascular risk groups were ischemic heart disease (56%), valve disease (43%) and hypertension (41%); for GPs these were hypertension (53%), ischemic heart disease (31%), and atrial fibrillation (23%) respectively [20].

### Use of the echocardiography service by the GPs

In our study, the GPs ordered an echocardiography cautiously. The low percentage of patients without echocardiographic abnormalities in the group 'other' indications' supports the view that GPs had a good judgment of the benefit of an echocardiogram requested for indications other than the three original indications. On the other hand, one might wonder whether GPs should not have used the open access service more often. On average, a GP ordered an echocardiographic examination for one patient per year. The incidence of heart failure in the Netherlands is 2 ‰ per year for men and 2.3 ‰ per year for women [21]. The standard list of patients per GP in The Netherlands is over 2000. According to these numbers, an average GP would establish a new diagnosis of heart failure in at least five patients per year, two men and three women. This diagnosis would be the result of a diagnostic process among patients in whom heart failure had been part of the differential diagnosis: patients older than 45 years of age, who presented with symptoms like dyspnoea, ankle oedema or fatigue, and in whom respiratory causes could be excluded. A rough estimate would be 20 'suspected' patients per GP per year. These patients can be subdivided into three categories. The first category includes patients in whom the diagnosis heart failure is evident from the patient's symptoms, medical history and physical examination. The second category includes patients who are already treated by a cardiologist the moment the diagnosis 'heart failure' is made. In both categories, the GP may consider an echocardiogram not necessary. The last category includes patients with non-conclusive symptoms who need an echocardiogram to confirm or exclude the diagnosis heart failure. These patients could benefit from an open access echocardiography service. Taking this into account, inclusion of roughly one patient per GP per year for open access echocardiography is probably lower than could be expected. Together with the relative small percentage of patients without abnormalities (23.5%) in comparison with other studies [18,22,23], this might imply GPs were too strict in their test ordering behaviour. Another option is that many patients who were suspected of having heart failure still were referred to the cardiologist while they would be suitable for the open access service.

The analysis over five years showed that GPs became less strict in their indications (more requests for 'other' indications), but also less efficient (i.e. a lower chance of pathological results of the echocardiographic examinations).

### Patient management

On average, the percentage of advice given by the cardiologist was low: in half of the cases with an echocardiographic abnormality. However, this figure improved over time (60% in recent cases vs. 40% in previous cases). Our study is one of the few to examine the management of the GP after receiving advice of the cardiologist. One study provided GPs with an interpretation of a scan and with guidance on management, but whether these recommendations were implemented was not described [4]. In another study was explained that one had chosen not to provide specific advice about management for fear that these recommendations might be implemented without considering other factors in their decision [6]. This worry probably is not justified: in our study, the GPs adhered to the advice to refer the patients to the cardiology outpatient department in only two-third of cases with echocardiographic abnormalities and GPs also referred patients for whom the cardiologist had not suggested to do so. From our results, one might deduce that GPs varied in their adherence behaviour depending on their reason for the request and the outcome. E.g., in patients referred because of a 'cardiac murmur' or 'peripheral oedema', the GPs followed the advice of the cardiologist more closely. In addition, when the outcome was 'valve disease' or 'LVD', GPs more often adhered to the cardiologist's advice.

### Implications for clinical practice

The purpose of starting the open access echocardiography service was to lower the threshold for supplementary diagnostic testing in patients suspected of heart failure. Our analysis demonstrates that with the current indications for open access echocardiography many patients with heart failure, especially diastolic heart failure, are detected. We reasoned however, that more patients could have been examined, taking the incidence of heart failure into account. Our study also revealed that GPs widened the indication spectrum for an echocardiographic examination with 'evaluation for possible LVH' in patients with hypertension. Given the high number of positive results, this seemed a justified choice. To improve the service in the future, we suggest evaluating the service regularly in joint meetings of GPs and cardiologists, to discuss potential adaptations of criteria for referral and wishes for the communication of results.

What diagnostic choices could a GP make in case of suspicion of heart failure? Some GPs will order an echocardiogram directly; others will first perform more accessible tests like an ECG or the measurement of the plasma concentration of (NT-pro)BNP. During the first years in which the open access service was operational, measurement of (NT-pro)BNP was not yet available. In previous studies the value of an ECG and measurement of (NT-pro)BNP was already pointed out as possible screening tools for excluding heart failure [1,8,19]. Recent studies do not agree yet about the value of a normal ECG to exclude heart failure [24,25]. There is growing consensus that a normal ECG is unlikely to be consistent with heart failure [26]. The same is true for a normal or low concentration of (NT-pro)BNP [26,27]. Considering the diagnostic value of both tests separately, it is plausible to assume that if both tests are normal heart failure can be excluded as a cause of the symptoms. If one of these two tests is abnormal, an echocardiogram could be requested.

What diagnostic choices could a GP make when he wants to evaluate the presence of LVH? This study from primary care showed a high number of patients with LVH, an important precursor of overt heart failure. Patients with LVH should be treated with RAAS-inhibitors to prevent or delay development to heart failure [28]. Therefore, it is important to detect patients with LVH who are not being treated yet with RAAS-inhibitors. Only these patients will benefit from further evaluation with echocardiography [29]. However, according to recent studies, a positive ECG is sufficient to prove the presence of LVH. In these cases, an echocardiogram would not be necessary [30]. But an ECG negative for LVH is not sufficient to exclude LVH. This implies that if a patient with hypertension is not treated with an RAAS-inhibitor yet, and has an ECG negative for LVH, an echocardiogram is appropriate to determine whether or not LVH is present [30].

## Conclusion

Among GPs, open access echocardiography is a popular service to detect patients with heart failure and patients at risk for developing heart failure. Overall, GPs used the open access echocardiography service efficiently (i.e. with a high chance of relevant pathology), but efficiency decreased slightly over the years. To meet the needs of the GPs, indications might be widened with 'suspicion LVH'. Further specification of the indications for open access echocardiography - by defining a stepwise diagnostic approach including ECG and (NT-pro)BNP - might improve the service and make it clearer for GPs when to use it.

## Competing interests

The authors declare that they have no competing interests.

## Authors' contributions

LB, RW, CLvdB and HS conceived and designed the study. LB, CLvdB, LvH, MT and MS collected the data. LvH, MT and MS participated also in data entry and data analysis. LB, HS and MT designed the questionnaire on GP management. LvH, under supervision of LB and HS, performed the statistical analysis. A draft version of the paper was made by LvH, partly based on earlier reports by MT and MS, and reviewed by all authors. HS and LvH wrote the final manuscript. All authors read and approved the final manuscript.

## Authors' information

LB and CLvdB are cardiologists at the 'Atrium Medisch Centrum Parkstad' Hospital in Heerlen, the Netherlands. LB is a honorary staff member of the Institute for Medical Education at the faculty of Health, Medicine and Life Sciences at Maastricht University Medical Centre. RW and HS are general practitioners in the ESL region, and both are associate professor at Maastricht University Medical Centre. LB, CLvdB, RW and HS initiated the Open Access Cardiology study as a research project for medical students in their final (6^th^) year. MT, MS and LvH are graduated physicians. At the time, they were medical students at Maastricht University Medical Centre, who had chosen this project for their research elective in year 6.

## Pre-publication history

The pre-publication history for this paper can be accessed here:

http://www.biomedcentral.com/1472-6963/10/37/prepub

## Supplementary Material

Additional file 1 **Translated Questionnaire on GP Management, Open Access Echocardiography Heerlen region**. English version of the Dutch questionnaire used to collect the data for the retrospective survey on management by the GP. Click here for file
