# Supplementary material for: Evaluation of an open access echocardiography service in the Netherlands: a mixed methods study of indications, outcomes, patient management and trends
Source: BMC Health Serv Res. 2010 Feb 10;10:37. doi: 10.1186/1472-6963-10-37 (PMC2835704; doi:10.1186/1472-6963-10-37)
Supplement: Additional file 1 — Translated Questionnaire on GP Management, Open Access Echocardiography Heerlen region. English version of the Dutch questionnaire used to collect the data for the retrospective survey on management by the GP. [file 1472-6963-10-37-S1.DOC]

**Translated Questionnaire on GP Management; Open Access Echocardiography Heerlen region**

When you considered requesting an echocardiogram at the Open Access Echocardiographic service, what were your considerations (more answers possible):

0 I wanted to confirm the diagnosis heart failure

0 I thought of a pulmonary cause of dyspnoea but wanted to exclude a cardiac cause

0 I didn’t know what was wrong at all

0 I heard a murmur but was not sure whether it was functional or caused by valve disease

0 A colleague (e.g. an occupational physician) heard a murmur and I wanted to have an assessment of this murmur

0 The patient was new in the doctor’s practice and was known to have a murmur or heart failure

0 The patient had hypertension and I wanted to exclude left ventricular hypertrophy

0 The patient had another condition (please mention which condition)

To confirm or exclude heart failure I performed the following investigations (several answers are possible):

0 Chest X-ray

0 Measurement of plasma BNP or NT-Pro-BNP

0 ECG

0 Spirometry

0 Diuretic test

0 Auscultation abnormalities:

0 Fourth heart sound

0 Third heart sound

0 Pulmonary rales

The patient was known to have the following conditions:

0 Diabetes mellitus or metabolic syndrome

0 Hypertension

0 Coronary insufficiency

0 COPD

0 Obesity

0 Other

I had already started the following medication:

0 Aspirin

0 Diuretics

0 ACE inhibitor / AT 2 inhibitor

0 Beta blocker

0 Nitrate

0 Calcium antagonist

0 Pulmonary medication

What did you do with the patient after receiving the results from the echocardiogram?

0 I started to treat the patient with

0 Aspirin

0 Diuretics

0 ACE inhibitor / AT 2 inhibitor

0 Beta blocker

0 Nitrate

0 Calcium antagonist

0 Pulmonary medication

0 I referred the patient to the cardiologist

0 I referred the patient to another medical specialist

0 I changed the medication

0 I reassured the patient
